# Genome Sequence of *Klebsiella pneumoniae* Strain ATCC 25955, an Oxygen-Insensitive Producer of 1,3-Propanediol

**DOI:** 10.1128/genomeA.00587-13

**Published:** 2013-08-08

**Authors:** Yu Wang, Fei Tao, Chao Li, Lixiang Li, Ping Xu

**Affiliations:** State Key Laboratory of Microbial Metabolism and School of Life Sciences & Biotechnology, Shanghai Jiao Tong University, Shanghai, China

## Abstract

*Klebsiella pneumoniae* strain ATCC 25955 is a 1,3-propanediol-producing bacterium that is insensitive to oxygen. Here, we present a 5.29-Mb assembly of its genome sequence. We have annotated 10 coding sequences (CDSs) for 1,3-propanediol fermentation and 18 CDSs for glycerol uptake. The CDSs related to virulence and by-product formation were also annotated.

## GENOME ANNOUNCEMENT

1,3-Propanediol (1,3-PD), a valuable bifunctional molecule, has several promising properties for many synthetic reactions, particularly for polymer synthesis (1). Polymers based on 1,3-PD, such as polytrimethylene terephthalate (PTT), have superior characteristics of stretching, stretch recovery, and better washfastness. Production of PTT has caused a drastic rise in demand for 1,3-PD (2). Recently, conversion of glycerol to 1,3-PD by microorganisms has drawn much attention, since a surplus of glycerol was produced as a by-product of the biodiesel industry (1).

A number of microorganisms can produce 1,3-PD from glycerol, such as strains of *Klebsiella*, *Clostridium*, and *Citrobacter* (3, 4). *Klebsiella pneumoniae* is one of the best natural 1,3-PD producers, not only for its appreciable yield and productivity, but also because of the availability of genetic engineering protocols (5). Metabolic pathways and enzyme kinetics of 1,3-PD production have been well studied, revealing that the first enzyme of glycerol fermentation, glycerol dehydratase, undergoes inactivation by O_2_ (5, 6). Therefore, anaerobic conditions are usually required for 1,3-PD production by *K. pneumoniae* (3, 7). Nevertheless, several *K. pneumoniae* strains, including strain ATCC 25955, are insensitive to oxygen, and they can convert glycerol to 1,3-PD under aerobic or microaerobic conditions, which are easier to handle in industrial applications (8). Previous studies indicated that strain ATCC 25955 could produce 73.3 g liter^–1^ of 1,3-PD with a yield of 0.48 mol mol^–1^ and a productivity of 1.5 g liter^–1^ h^–1^ (9). The main by-product is 2,3-butanediol (BD), and our primary tests suggested that three stereoisomers of BD were produced from glycerol by strain ATCC 25955 (data not shown). To eliminate by-product formation and pathogenicity, genetic modification of *K. pneumoniae* is desirable. Genome sequencing of *K. pneumoniae* ATCC 25955 will be of great help in this regard.

Here we present the first draft genome sequence of strain ATCC 25955, which we determined by using the Illumina HiSeq 2000 system. The reads were assembled into 88 contigs by using Velvet (10). The genome annotation was performed by use of the RAST server (11). The G+C content was calculated using the genome sequence.

The draft genome sequence includes 5,290,220 bases and is comprised of 5,007 predicted coding sequences (CDSs) and 83 RNAs, with a G+C content of 57.4%. According to the annotation, we have predicted 10 CDSs responsible for glycerol fermentation to 1,3-PD. The glycerol dehydratase, 1,3-PD dehydrogenase, and the glycerol dehydratase reactivation factor of strain ATCC 25955 differ in several amino acids from those of strain *K. pneumoniae* LZ (7), an anaerobic 1,3-PD producer. Further investigation of these genes may help to demonstrate the mechanisms of 1,3-PD fermentation. Although (2*R*,3*R*)-BD was detected in the broth, no (2*R*,3*R*)-BD dehydrogenase-encoding genes were annotated. Moreover, there are 18 and 140 CDSs related to glycerol uptake and to virulence, disease, and defense, respectively, which should be investigated for use in eliminating by-product formation and pathogenicity.

### Nucleotide sequence accession numbers.

The whole-genome shotgun project has been deposited at DDBJ/EMBL/GenBank under the accession number AQQH00000000. The version described in this paper is the first version, AQQH01000000.
